# A 454 multiplex sequencing method for rapid and reliable genotyping of highly polymorphic genes in large-scale studies

**DOI:** 10.1186/1471-2164-11-296

**Published:** 2010-05-11

**Authors:** Maxime Galan, Emmanuel Guivier, Gilles Caraux, Nathalie Charbonnel, Jean-François Cosson

**Affiliations:** 1 INRA EFPA, UMR CBGP (INRA/IRD/Cirad/Montpellier SupAgro), Campus international de Baillarguet, CS 30016, F-34988 Montferrier-sur-Lez cedex, France; 2 Laboratoire d'Informatique de Robotique et de Microélectronique de Montpellier (LIRMM) CNRS: UMR5506 - Université Montpellier II - Sciences et Techniques du Languedoc, France; 3 Montpellier SupAgro, 2 Place Pierre Viala, F-34060 Montpellier, France

## Abstract

**Background:**

High-throughput sequencing technologies offer new perspectives for biomedical, agronomical and evolutionary research. Promising progresses now concern the application of these technologies to large-scale studies of genetic variation. Such studies require the genotyping of high numbers of samples. This is theoretically possible using 454 pyrosequencing, which generates billions of base pairs of sequence data. However several challenges arise: first in the attribution of each read produced to its original sample, and second, in bioinformatic analyses to distinguish true from artifactual sequence variation. This pilot study proposes a new application for the 454 GS FLX platform, allowing the individual genotyping of thousands of samples in one run. A probabilistic model has been developed to demonstrate the reliability of this method.

**Results:**

DNA amplicons from 1,710 rodent samples were individually barcoded using a combination of tags located in forward and reverse primers. Amplicons consisted in 222 bp fragments corresponding to *DRB *exon 2, a highly polymorphic gene in mammals. A total of 221,789 reads were obtained, of which 153,349 were finally assigned to original samples. Rules based on a probabilistic model and a four-step procedure, were developed to validate sequences and provide a confidence level for each genotype. The method gave promising results, with the genotyping of *DRB *exon 2 sequences for 1,407 samples from 24 different rodent species and the sequencing of 392 variants in one half of a 454 run. Using replicates, we estimated that the reproducibility of genotyping reached 95%.

**Conclusions:**

This new approach is a promising alternative to classical methods involving electrophoresis-based techniques for variant separation and cloning-sequencing for sequence determination. The 454 system is less costly and time consuming and may enhance the reliability of genotypes obtained when high numbers of samples are studied. It opens up new perspectives for the study of evolutionary and functional genetics of highly polymorphic genes like major histocompatibility complex genes in vertebrates or loci regulating self-compatibility in plants. Important applications in biomedical research will include the detection of individual variation in disease susceptibility. Similarly, agronomy will benefit from this approach, through the study of genes implicated in productivity or disease susceptibility traits.

## Background

Highly polymorphic genes constitute a major component of the functional genetics of biota. They are involved in many crucial functions including the regulation of self incompatibility in plants [1,2], fungi [3] and marine invertebrates [4], immunity in vertebrates [5,6] or insects [7], sex-determination in insects [8,9] and disease resistance in plants [10]. By definition, such genes display a very high number of alleles/variants within a single population or a single species, and many individuals are heterozygous for these genes. For instance, exon 2 of the MHC class II gene *DRB *has 878 variants in humans and displays an excess of heterozygotes within populations IMGT/HLA database,[11]. Another feature is their mode of evolution, promoting interspecific polymorphism MHC, [12]. This makes them particularly interesting in the study of community genetics e.g. [13,14]. Furthermore, the potential involvement of highly polymorphic genes in inter-specific interactions outcomes may promote the emergence/persistence of biodiversity through speciation or diversifying selection processes [14-17].

Despite their important roles in genetics and evolution, in the context of medicine or agronomy, highly polymorphic genes are seldom studied within population and community genetics. In non-model organisms, one major limitation is the difficulty of genotyping high numbers of individuals. Highly polymorphic genes are, by definition, prone to display numerous, extremely diverse variants as well as many heterozygotes within populations, which can hardly be genotyped using direct sequencing. The direct sequencing of amplicons would often result in the superposition of two very different sequences, giving mostly unreadable electropherograms. Sequencing must therefore be preceded by the separation of the two copies (for diploids) of the gene. For the classical Sanger method, a previous step of cloning is necessary, which is expensive and time consuming. Alternative methods rely on the indirect characterisation of sequence variability and involve capillary electrophoresis single-strand conformation polymorphism (CE-SSCP), denaturing gradient gel electrophoresis (DGGE), high resolution melting curve analysis (HRMCA), PCR using sequence-specific primers (PCR-SSP), oligonucleotide chips or other related techniques see e.g. [18-23]. However, these indirect methods are not fully informative because of the non-negligible rates of homoplasy, i.e. preventing different variants from being distinguished based on sequence conformation e.g. [24]. Moreover, they do not provide the nucleotide sequences, thus precluding many analyses in evolutionary and functional genetics. Cloning the target sequences into bacteria and sequencing different clones using the Sanger approach, so as to recover the different variants, may be required to further analyze relationships between migration patterns and sequences e.g. [25]. Such approaches are expensive and time consuming and require many clones to be sequenced for each sample in order to guarantee a high probability of including all variants e.g. [26]. Cloning-sequencing is often unaffordable for population and community studies, which require several hundreds of individuals to be genotyped.

Over the last five years, the development of high-throughput genomic sequencing technologies has opened up new and exciting perspectives in evolutionary studies, biomedicine and agronomy [27]. The 454 GS FLX (Roche) platform, for instance, allows the reading of 100 bp- to 500 bp-fragments. Unlike the classical Sanger method, the 454 technology includes an emulsion polymerisation chain reaction (emPCR) before the pyrosequencing step [28]. This stage allows the isolation of each DNA strand before sequencing, just as in the cloning-sequencing approach. This feature is of particular interest in the characterization of genetic variability of single highly polymorphic and multi-copy genes, for which many very different variants may co-occur within individuals. The main limitation of the 454 methods remains the high cost of each run (between 7,000 and 20,000 euros). This cost is, however, compensated by the high number of reads produced in one run [> 1 million reads (see Table 1 for definition), [29]]. It is thus theoretically possible to genotype a high number of individuals. Such large-scale pyrosequencing of genes has been applied to detect SNPs and small deletions and insertions: for example those potentially involved in hereditary diseases and cancers [30]. However, the genetic variation observed in the mentioned study was not reattributed to original samples. Large-scale pyrosequencing is thus a promising approach, provided that each read produced may be reliably attributed to its original sample. In this way, Babik *et al*. [31] have applied the 454 technology to genotype 96 individuals at the MHC class II *DRB *gene. Different solutions have been proposed recently to allow *a posteriori *attribution of the sequences produced. One straightforward way to recover the original sample of a given sequence is to use nucleotide barcodes. They consist in short nucleotide sequences called 'tags' fixed at the extremity of DNA strands. These tags must produce a unique barcode for each sample. Those are either ligated to DNA fragments to be sequenced [32], or are included directly in the 5' end of the primers for sequencing amplicons (i.e. tagged-primer in [33]). For both approaches, the number of different tags to be synthesized is equal to the number of samples to be genotyped. By contrast, the method designed by Bierne *et al*. [34] for cloning-sequencing studies is based on the combination of tags in the forward and reverse primers. Only *n *sets of primers are thus required for the coding of *n*^2 ^samples. This approach has recently been applied to 454 sequencing system [35].

**Table 1:** Definition of the terms used.

Term used	Definition
Reads	Sequences passing quality control (QC) criteria after BaseCall, generated from 454 sequencing using manufacturer specifications.
Sequences	Reads remaining in the dataset after the first step of our data processing procedure (Table 2).
Variants	Sequences differing by at least one base pair substitution or by an indel.
Artifactual sequences or artifactual variants	Sequences or variants that resulted from sequencing errors, polymerase errors and non-specific amplifications of paralogue and pseudogene during PCR (Table 2).
True sequences or true variants	Sequences or variants that were retained after validation at all stages of our stepwise procedure.

Here we describe a 454 approach that shows similarities with the one developed by Babik *et al*. [31], but that is optimized for the analysis of thousands of individuals in a single region of a 454 plate. Using one half of a 454 run, we were able to barcode PCR amplicons from 1710 samples of rodents, corresponding to 24 species. We used combinations of tagged primers, for a nuclear gene that is known to be highly polymorphic in mammals, the MHC class II DRB gene for a review see [36]. Amplicons were multiplexed for the emPCR to produce a high number of reads in a single run. The tagged primers also allowed *a posteriori *attribution of most reads to their sample of origin. We then proposed a stepwise procedure for data analysis and variant validation. Sequences containing errors have previously been shown to occur in some reads during 454 sequencing [37]. Those needed to be distinguished from correct sequences. We developed a probabilistic model to provide a confidence level for each genotype observed. This model will also be useful for optimizing the number of samples to be multiplexed in one 454 run.

## Methods

### Samples and DNA extractions

The experiment was based on 1710 rodent tissue samples (either toes or lungs) collected between 2001 and 2008 in Europe, Southeast Asia and Caribbean islands. These samples corresponded to 1614 individual rodents belonging to 24 different species and 11 genera. Reproducibility of the genotyping was estimated for 96 samples that had been randomly chosen from the dataset and processed twice for the entire procedure.

DNA was extracted from the 1710 samples using 18 plates of the DNeasy Tissue Kit (Qiagen) following the manufacturer's recommendations. Various steps of the experiment were carried out using rigorous laboratory protocols to prevent contamination by alien DNA and amplicons. PCR plates were prepared in a DNA-free room and under a sterile hood. We systematically used filter tips for the different steps of DNA extraction and PCR. The absence of contamination was checked at this stage and along the whole laboratory procedure using three negative controls per extraction plate. They corresponded to one extraction blank (extraction without sample tissue), one PCR blank (a tube with PCR mix without DNA) and one aerosol blank (a PCR blank with the cap open during the whole manipulation).

The total DNA set was then divided into two sets of 855 DNA samples, referred to as Pool A and Pool B. These pools were independently analyzed using the same combination of barcodes. This allowed the relative efficiency of the different barcodes to be determined.

### Tagged primer design, PCRs and sequencing

**Figure 1:**
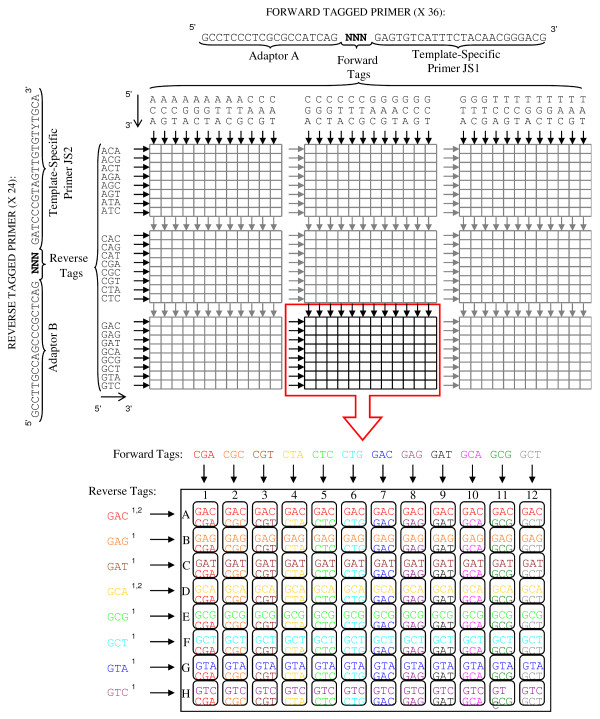
**Design of the barcoding system**. 36 forward and 24 reverse tags were dispensed into nine 96-well plates producing 864 unique combinations of 6-nucleotide sequences named barcodes. ^1^: reverse tags forming a homopolymer GG with the "key" sequence. ^2^: reverse tags that could not be recovered because of homopolymer formation (see text for more details).

We used the target-specific primers developed by Schad *et al*. [38] to analyze exon 2 of the *DRB *gene in rodents. These primers are JS1 (Forward 5'-GAGTGTCATTTCTACAACGGGACG-3') and JS2 (Reverse 5'-GATCCCGTAGTTGTGTYTGCA-3'). They amplify a 171 bp fragment (excluding primers) of exon 2 from the *DRB *gene in several mammal species [38]. One or several copies can be amplified with this set of primers depending on the rodent species considered (e.g. a single copy in *Apodemus *sp. [39], duplicated copies in *Gerbillurus paeba *[40] and *M. glareolus *[41]). There was no prior knowledge on *DRB *variability and duplication for all species studied except *M. glareolus *and *Apodemus sp*. in our experiment. These primers were modified by adding a short 3 bp sequence (the tag) and 19 bp adaptors to the 5' ends of JS1 ('A': 5'-GCCTCCCTCGCGCCATCAG-3') and JS2 ('B': 5'-GCCTTGCCAGCCCGCTCAG-3'). These adaptors were required for the emPCR and 454 pyrosequencing (see Figure 1). The key sequence TCAG at the 3' ends of both adaptors A and B, which were used during the BaseCall step as a quality control (QC) measure to validate the reads. Primers were synthesized by Eurogentec and purified using the standard selective precipitation optimized process (SePOP).

We designed 36 different tags for the forward primers, and 24 different tags for the reverse primers (see details in Figure 1). This leads to 864 potential unique combinations of forward and reverse tags. Samples were individually barcoded during the preparation of the PCR plates. Thirty-six forward primers were dispensed into the plates 'vertically', so that each row contained a different forward primer, while the 26 reverse primers were dispensed 'horizontally', with wells in each column containing a different reverse primer (Figure 1). Thus, each well harboured a single, unique 6-bp barcode through the combination of forward and reverse tags.

PCRs were carried out in a 25 μL reaction volumes containing 2 mM MgCl2, 100 μM dNTPs, 0.1 μM of each primer and 0.75 U AmpliTaq Gold^® ^(Applied Biosystems) in the appropriate 1× PCR Buffer II and de-ionized DNA- and RNA-free water. DNA samples were added to this reaction mix in a separate room to avoid DNA contamination. DNA (1.5 μL, i.e. nearly 40 ng) was added to each well. The PCR was optimized through touchdown protocol. Samples were subjected to initial denaturation at 95°C for 15 min, followed by 10 cycles of denaturation at 94°C for 30 s, annealing with touchdown at 65°C to 55°C (-1°C/cycle) for 30 s and extension at 72°C for 30 s, followed by 30 cycles of denaturation at 94°C for 30 s, annealing at 55°C for 30 s and extension at 72°C for 30 s, with a final extension phase at 72°C for 10 min. PCR products (5 μL) were checked on a 1.5% agarose gel. The remaining PCR products were sent to Cogenics™ Genome Express where pyrosequencing was carried out using a 454 GS FLX System (Roche Diagnostics GmbH/454 Life Sciences Corporation).

All PCR products were analyzed using the LabCHIP^® ^90 system and DNA CHIP 5 K from Caliper Life Science. This microfluidic electrophoresis allows fragment size and individual amplicon concentration, excluding primer and dNTP residues, to be determined. As described above, two equimolar pools (Pool A and Pool B) of amplicons were produced. To eliminate unspecific products, the two pools were run on an agarose gel and purified by gel excision of fragments of 260 bp ± 12 bp. The quality of the pools were checked by size range analysis using a DNA 1000 Assay on the 2100 Bioanalyzer (Agilent Technologies) and quantified by fluorescent measurement using the Quant-it™ Picogreen^® ^DNA assay (Invitrogen).

The emPCR, corresponding to clonal amplification of the purified amplicon pool, was carried out using the GS emPCR Kit II (Roche Diagnostics GmbH). Briefly, amplicons were immobilized onto DNA capture beads. The amplicon-beads obtained were added to a mixture of amplification mix and oil and vigorously shaken on a Tissue Lyser (Qiagen) to create "micro-reactors" containing both amplification mix and a single bead. Emulsion was dispensed into a 96-well plate and the PCR amplification program was run according to the manufacturer's recommendations. Following amplification, the emulsion was chemically broken and the beads carrying the amplified DNA library were recovered and washed by filtration. Positive beads were purified using the biotinylated primer A (complementary to adaptor A), which binds to streptavidin-coated magnetic beads. The DNA library beads were then separated from the magnetic beads by melting the double-stranded amplification products, leaving a population of bead-bound single-stranded template DNA fragments. The sequencing primer was then annealed to the amplified single-stranded DNA. Lastly, beads carrying amplified single-stranded DNA were counted with a Z2™ Cell Counter (Beckman Coulter).

Pools A and B were simultaneously processed in one GS FLX run using one half of a 70 × 75 mm Pico-Titer plate device (Roche Diagnostics GmbH) and one GS LR-70 sequencing kit (Roche Diagnostics GmbH). Briefly, the 70 × 75 mm Pico-Titer plate was divided in four sections (one corresponding to Pool A, one to Pool B and two to another pyrosequencing project) using the Medium Regions Bead Loading Gasket (Roche Diagnostics GmbH). According to the manufacturer's recommendations for amplicon sequencing, 250,000 DNA beads were loaded for each pool mixed with an appropriate volume of packing beads and enzyme beads. After the Pre-Wash run, the sequencing run was started with the "Full Analysis for Amplicon" parameter set.

### Bioinformatics and data processing

A non-negligible number of errors are generated during PCR [42] and pyrosequencing [37]. Non-specific amplification may also occur. We thus developed a stepwise data processing procedure aimed at detecting and discarding most of the reads that exhibit sequencing errors or that correspond to genes other than the targeted gene (see objectives and rationales, Table 2). This procedure relied on three assumptions. First, reads with errors were expected to appear at lower frequencies than reads without errors in the whole dataset and for each individual sample. This assumption was validated using a probabilistic approach. Considering the probability of substitution errors, the probability of artifactual variants (that arose from substitution errors) occurring in our dataset was ≈ 0.11, and the probability of observing three times the same artifactual variant is very low, *p*_1 _≈ 10^-8 ^(Additional Information [see Additional file 1]). Second, reads exhibiting indels with lengths that are not multiples of three base pairs were considered as artifacts because they would induce shifts in the reading frame. Third, the reliability of the genotype obtained for a given individual sample should depends mainly on the number of sequences obtained for this sample and on the number of copies of the gene in the species studied.

**Table 2:** Objectives and rationales for each step of the data processing procedure to validate variants and genotypes.

Steps	Objectives	Rationales
1	Remove reads with incomplete barcode information.	Reads cannot be assigned to any individual.
	Remove reads that display imperfect match with the primers.	An imperfect match with primers may cause a shift in the reading frame and/or errors in the barcode.
	Remove sequences that are unique within the pool.	Unique sequences probably result from sequencing errors (first assumption). This step reduced the dataset and then facilitated subsequent data analyses. Note that this step may be relaxed for small datasets (unique sequences will also be removed during Step 3).
	Remove reads that display indels that are not multiples of three base pairs.	Such indels probably result from sequencing errors (second assumption). Note that this step may be relaxed when focusing on non-functional genes.

2	Remove samples with a low number of sequences.	A low number of sequences may induce an incomplete genotyping (second assumption). The minimum number of sequences required to obtain a reliable genotype is estimated taking into account the number of copies amplified for the gene studied (threshold 1).

3	Remove variants with a low number of sequences for a given sample.	Variants represented only rarely within samples probably result from sequencing errors (first assumption). The minimum number of sequences required to validate a given variants of an individual genotype is estimated from the distribution of variant frequencies for the given sample (threshold 2).

4	Remove variants that do not correspond to the gene studied, using sequence alignment.	Some inconsistencies may still exist in the dataset such as recombinant chimeric sequences originating from a mixture of the sequences of two different alleles, [42], pseudogenes or paralogs, which can occur at high frequencies within individual samples.

See text for the definitions of assumptions and thresholds.

Our validation procedure involved four consecutive steps (Table 2). Briefly, the first cleaning step allowed removal of most imperfect reads from the dataset (reads with incomplete primers or barcodes, reads corresponding to sequences that were observed only once in the entire dataset and reads exhibiting indels of sizes that were not multiples of three base pairs). The next two steps were based on thresholds under which the number or frequency of sequences obtained per sample was insufficient to provide reliable genotypes. Rationales and procedures for establishing these thresholds are described below. The last step involved the alignment of remaining sequences using Mega 4.0 [43]. This step allowed the detection and elimination of sequences that corresponded to pseudogenes, paralogs and recombinant chimeric sequences originating from a mixture of sequences of two different variants e.g. for chimeric sequences produced during PCR see [42]. Paralogs and pseudogenes were then identified using nucleotide blast against all sequencing data available in Genbank (default parameters).

### Basic statistics and genetic diversity

We calculated simple statistics for the whole dataset and independently for pools A and B. Statistical parameters took into account the total number of reads (*R*), the total number of sequences (*N*), the total number of variants (*A*) and the number of sequences obtained for each variant *j *(*N_j_*). These same statistical principles were applied for each individual sample *i *as the total number of sequences (*N_i_*), the number of variants (*A_i_*), the number of sequences for each variant *j *(*N_ij_*) and the frequency of each variant (*F_ij_*), corresponding to *N_ij_/N_i_*.

### Thresholds for genotype and variant validation

We then determined a confidence level for each genotype. The rationales for this was that (i) a minimal number of sequences are required for reliable genotyping; and (ii) true variants must be sequenced several times within the same sample in order to be validated and distinguished from artifactual variants (Table 1). We first defined the threshold *T_1_*, corresponding to the minimal number of sequences required per individual to determine its complete genotype, with a low probability of missing variants. We computed the probability of amplifying at least *r *times all the variants of the gene studied for a given sample. This probability was based on *n*, the total number of true sequences obtained for the sample, and *m*, the maximal number of variants for the gene within a sample. The value *m *depends on the number of gene copies and on the degree of ploïdy of the studied organism. For instance, *m *= 2 for a single copy of a nuclear gene within a diploid genome, and *m *= 4 for a duplicated nuclear gene within a diploid genome, or for a single-copy nuclear gene in a tetraploïd genome, and so on. A program is now freely available on the web http://www.lirmm.fr/~caraux/Bioinformatics/NegativeMultinomial/ to calculate the probability *f(r,m,n) *of having at least *r *sequences of each of the *m *variants potentially observed within the *n *sequences of a given sample. Here, we considered and discussed different values for *r *(1, 3, 5 and 10) and for *m *(2, 4, 6 and 8).

We then defined a second threshold, *T_2_*, to eliminate artifactual variants that arose from substitution errors. Based on our initial assumptions and in accordance with the results of our probabilistic calculations (Additional Information [see Additional file 1]), artifactual variants should occur at much lower frequencies than true variants. We therefore analyzed the distribution of variant frequencies calculated individually for each sample (*F_ij_*, see above). We expected multinomial-shaped distributions with one peak at very low frequency values corresponding to artifactual variants and several peaks at medium-high frequency values corresponding to true variants. These peaks should include a very high frequency peak (about 90-100%), corresponding to homozygous samples, and one or more peaks of medium frequency values (30 to 50%), corresponding to heterozygous samples. Ideally, the threshold value *T_2 _*should be chosen to discard most of the artifactual variants and none (or very few) of the true variants.

### Validation and efficiency of the method

We analyzed the efficiency of barcoding by comparing the number of sequences obtained for each tag between pools A and B. We used a one way ANOVA with the number of sequences as an independent variable, and the reverse tag, the forward tag and the pool as dependent qualitative variables. All two-way interactions were tested and removed when not significant. This analysis was performed on the dataset obtained after the first step of the data processing. The number of sequences was log-transformed to normalize its distribution. *Post-hoc *pairwise comparisons were performed with the Tukey-Kramer method. We then compared the genotypes obtained for the 96 replicated samples to investigate the reproducibility of the genotyping. Finally, we examined the distribution of the frequency of true and artifactual variants within samples (*F_ij_*). All analyses were performed using GENSTAT 7.1 (Lawes Agricultural Trust, Rothamstead).

## Results

None of the 54 blanks were found to be positive after PCR amplification. We obtained 221,789 quality control (QC) reads overall (*R*), with *R*_A _= 91,467 for pool A and *R_B _*= 130,322 for pool B. This difference between pools was probably attributed to the difference in DNA concentration between pool purifications, which was 0.83 and 1.84 ng/μL for pools A and B, respectively.

### Data processing

#### Step 1

The first step was the detection and suppression of most of the imperfect reads (Table 2). We had some difficulties in determining sequence using reverse tags starting with base G. These tags formed a homopolymer GG with the "key" sequence (e.g. 5'-TCA**G**-**G**NN-3'). In this case it appears that the G of the tag was removed altogether with the key sequence during the data processing with the GS-FLX Data Analysis Software, creating a shift of one base in tag and sequence reading. This problem was encountered for eight reverse tags (see Figure 1). We performed an *ad hoc *analysis, focusing on the two last bases of the tags, allowing sequence information to be recovered for six of the eight tags. After the elimination of imperfect reads, the number of sequences per variant displayed an L-shaped distribution (Figure S1 [see Additional file 2]). The number of reads that could be assigned to variants in each pool were 63,841 and 89,508, corresponding to 10,420 and 9,427 variants, in pools A and B, respectively. We found 7,686 variants (74%) and 5,985 variants (64%) to occur only once in datasets A and B, respectively. The minimum and maximum numbers of sequences assigned per variant were 1 and 2,513 sequence(s) per variant in pool A, and 1 and 12,688 in pool B. Variants occurring only once and variants containing indels were then removed from both datasets. This considerably reduced the datasets and facilitated further bioinformatic manipulations.

At the end of the first step, we had *N_A _*= 53,661 and *N_B _*= 81,627 sequences, corresponding to *A_A _*= 2,322 and *A_B _*= 2,833 variants for pools A and B, respectively. Altogether, about 33% of the reads were eliminated in the first step. Over the 855 samples studied in each pool, we succeeded in assigning sequences for 745 of them in pool A and 764 in pool B. The number of sequences obtained for each sample strongly depended on the sample considered (Figure S2 [see Additional file 2]). Averages of sequences per sample were respectively *N_Ai _*= 65.6 (min. = 0, max. = 207) and *N_Bi _*= 82.7 (min. = 0, max. = 297) for pools A and B. Overall we did not obtain any sequence for 201 samples. This was explained for 54 samples by low amplicon concentrations observed before pooling (< 1 ng/μl) and probably resulting from poor DNA conservation. For the 144 other samples, the reverse tag sequence could not be recovered because of the formation of a homopolymer with the sequencing key (see above).

#### Step 2

The aim of the second step was to provide a confidence level for each genotype produced. We estimated the probability of amplifying at least *r *times all the different variants of the gene studied for a given sample. This probability depended on *n*, the total number of true sequences obtained for the sample, and *m*, the maximal number of variants for the gene within a sample (see the Materials and Methods section). We then plotted the probability *f(r,m,n) *against the number of sequences *n *for different values of *r *and *m *(Figure S3 [see Additional file 2]). As expected, we observed that for a given probability, *n *must be increased when *r *and *m *values increase. That means that to achieve a given confidence level, more sequences per sample (*n*) are required when the number of copies amplified of the gene under study (*m*) increases, or when the number of sequences required for validating an variant as true (*r*) increases. To further analyze our results, we fixed the *r*-value to three. This value was based on statistical considerations. Actually, we showed that the probability of observing three times the same artifactual sequence was very low, *p*_1 _≈ 10^-8 ^(see Additional Information [Additional file 1]). Moreover it also corresponded to the standard procedure in MHC gene studies where three independent observations of the same sequence are recommended to validate a variant that has been identified through a cloning-sequencing approach see [44]. Furthermore, our approach allows choosing any other value of *r *and x, depending on particular characteristics (e.g. number of samples and number of sequences for each sample) in other experiments.

**Figure 2:**
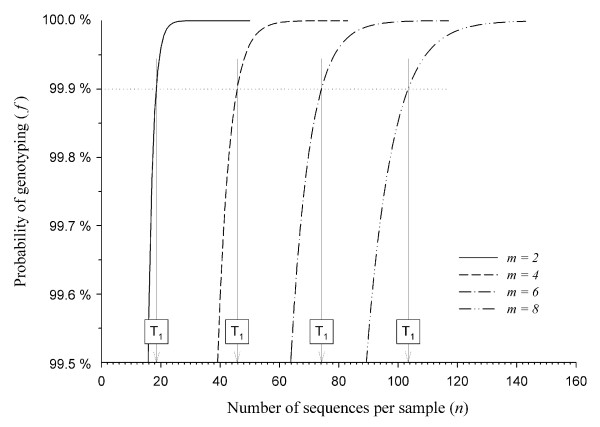
**Confidence level of genotyping**. *f *is the probability of amplifying, at least three times, all the different variants of the gene studied for a given sample. This probability depends on *n*, the total number of sequences per sample, and *m*, the maximal number of variants for the gene within a sample. *T_1 _*is the threshold value that corresponds to the minimal number of sequences required per individual to determine a complete genotype, with a 10^-3 ^probability of missing a variant.

The threshold *T_1 _*(i.e. the minimal number of sequences required for reliable genotyping) was then calculated for *r *= 3, giving a confidence level of 10^-3 ^(Figure 2). Simulations showed the threshold value *T_1 _*ranged from *T_1 _*= 19 for a single copy gene in diploid species to *T_1 _*= 104 for an octaploid species, or for a quadruplicated gene in a diploid species. In a similar way, *T_1 _*= 46 for a single copy gene in tetraploid species or for a duplicated gene in a diploid species. Samples with a number of sequences *n *<*T_1 _*were then removed from the dataset, as their genotypes were not considered to be reliable. At this stage, 75 samples were removed from the dataset, corresponding to 5% of the samples.

#### Step 3

**Figure 3:**
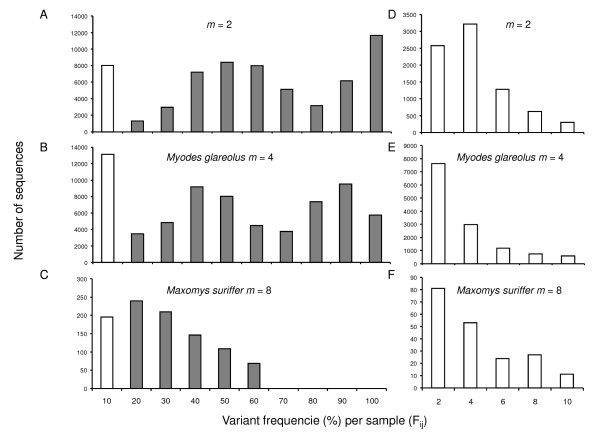
**Histograms showing the distributions of *F_ij_*, the frequency of each variant *j *within each individual sample *i***. Data were grouped as a function of *m*, the maximal number of variants for the gene within a sample: (A) for *m *= 2, (B) for *m *= 4 and (C) for *m *= 8. Histograms on the right display the distributions for values below ten percent: (D) for *m *= 2, (E) for *m *= 4 and (F) for *m *= 8.

The third step involved separating true from artifactual variants within each sample, based on the frequency of variants within samples (*F_ij_*). We plotted the distribution of *F_ij _*after grouping data based either on the number of copies of the *DRB *gene (Figure 3) or on taxonomy (Figure S4 [see Additional file 3]). All distributions obtained displayed the expected multimodal shape, with three peaks corresponding to frequencies of 1-4%, 30-60% and 80-100%. A few exceptions were detected, however. *Maxomys*, in which the *DRB *gene is quadruplicated, showed no variants in the 80-100% range, consistent with the absence of homozygotes. By contrast, *Niviventer *showed a low occurrence of variants in the 30-60% range, associated with a high occurrence of homozygotes. For the other species, we fixed the threshold *T_2 _*value to 4%. All variants with *F_ij _*<*T_2 _*in a given sample *i *were removed from the sample *i*, being considered as artifactual within the particular sample. By the end of this step, 48,294 sequences, corresponding to 600 variants, were conserved for pool A and 68,413 sequences, corresponding to 354 variants, were conserved for pool B. This step allowed the mean number of variants per sample to be reduced from 12 to 2.5, suggesting that most of the artifactual variants had been removed from the dataset.

#### Step 4

The sequences of the remaining variants were aligned separately for each sample, and analyzed by eye (Figure S5 for illustration [see Additional file 3]). We detected pseudogenes that were orthologous to the *RT1-Hb *gene of the laboratory rat *Rattus norvegicus*. We also found variants that were orthologous to the *DQB *gene, a paralog of the *DRB *gene, belonging to the same MHC class II multigenic family. Recombinant chimeric sequences were quite common in the dataset, occurring at high frequencies (up to 10%) in some samples. They were easily detected in alignments because their sequences originated from a mixture of the sequences of two different, true variants occurring in the same sample. After removing those non-specific variants from the data set, the number of sequences became lower than *T_1 _*(see Step 2) for 27 samples. Those samples were then eliminated, as their genotypes were not considered to be reliable.

At the end of our data processing procedure, 1407 samples were reliably genotyped (following our criteria) over the 1710 initially processed (201 samples removed at Step 1, 75 at Step 2 and 27 at Step 4). Moreover, 45,038 sequences and 251 variants had been retained in pool A, as where 65,836 sequences and 147 variants in pool B. Overall, 392 different variants were characterized and sequenced, with an average of 277 sequences per variant (min: 5; max: 12,688). Of the 392 variants finally validated by our data processing for exon 2 of the *DRB *gene, 36 had been previously reported in GenBank (*Myodes*: 15; *Arvicola*: 12; *Apodemus*: 8; *Rattus*: 1).

### Barcode bias

The ANOVA conducted on the numbers of sequences remaining after step 1 (i.e. after removing tags affected by the homopolymer problem) was significant (*F_112,1394 _*= 2.857, *P *< 10^-4^). The interaction between the two factors, "forward tags" and "reverse tags", could not be statistically tested in the ANOVA. The analysis showed significant differences between pools (*P *= 3 × 10^-4^) and between forward tags (*P *< 10^-4^). The use of forward tags CAC and CGC resulted in lower numbers of sequences, whereas TCA, TCT and TGT led to higher numbers of sequences (Figure S6 [see Additional file 2]). Interactions between pools and tags were significant and showed that differences between tags were not consistent between pools (Forward tags, *P *= 6 × 10^-3^; Reverse tags, *P *< 10^-4^). The lower numbers of sequences obtained for the forward tags CAC and CGC were only significant in pool B. Among reverse tags, GCT was associated with the lowest number of sequences in pool A, whereas CAC had the lowest number of sequences of all the other tags in pool B. Thus, despite finding some significant differences, no systematic bias could be related to tag sequences.

### Genotype reliability

Among the 96 replicated samples, only 66 were successfully genotyped twice by the end of our bioinformatics procedure. Unfortunately, the 30 other samples could not be genotyped twice due to the formation of homopolymers by particular tags (see above). Of these 66 samples, 63 displayed the same genotype in both replicates. The three samples showing non-identical genotypes were heterozygous for one replicate and homozygous (with a drop in one of the two variants) for the other. In one case, the 'missing' variant had been amplified, but its frequency within the sample (2%) was lower than the *T_2 _*threshold value (4%). Overall, the reproducibility of our genotyping was 95%.

Further analysis allowed the examination of 12,022 sequences, corresponding to 953 variants. True variants accounted for 84 ± 8% (mean ± S.E.%) of the sequences produced within samples, substitutions (1 or 2 bp) accounted for 6 ± 5%, insertions (1 or 2 bp) for 2 ± 3%, deletions (1 or 2 bp) for 4 ± 9%, chimeras for 6 ± 7% and other genes (pseudogenes and paralogs) for 1 ± 2%.

### Efficiency of the method

We were able to genotype 1,407 rodent samples from 24 different species at a confidence level of 10^-3^. *Maxomys *and *Niviventer *genera showed the lowest genotyping success rate (18% and 56%, respectively; Table 3). The quadruplication of the *DRB *gene in *Maxomys *(up to 7 variants were observed in some individuals) may account for most of the difficulties in genotyping these animals. However, *Niviventer *seemed to have a high occurrence of null alleles, with many individuals producing only a small number of sequences, and most other individuals being homozygotes (Table 3). The genotyping success rate was fairly high, generally above 90%, for all other species.

**Table 3:** Genetic variation in exon 2 sequences of the *DRB *gene observed for 1,407 rodent samples from 24 species and 11 genera.

Rodent Genera	Number of *DRB *copy amplified	Number of analysed samples	Replicates		Genotyped (*p *= 0.999)		Heterozygotes genotyped		Number of variant	Mean number of sequences per variant
			Nb	%	Nb	%	Nb	%		
Pool A										
*Myodes*	2	650	33	5.1	602	93	495	82.2	71	834
*Arvicola*	1	49	2	4.1	49	100	36	73.5	13	324
*Microtus*	1	40	2	5.0	36	90	29	80.6	7	403
*Apodemus*	1	44	3	6.8	42	95	39	92.9	50	70

Pool B										
*Rattus*	1	417	19	4.6	388	93	248	63.9	110	233
*Mus*	1	147	8	5.4	134	91	110	82.1	53	171
*Niviventer*	1	78	5	6.4	44	56	9	20.5	15	172
*Bandicota*	1	58	4	6.9	51	88	39	76.5	21	154
*Berylmys*	1	37	4	10.8	36	97	25	69.4	19	122
*Leopoldamys*	1	24	1	4.2	21	88	14	66.7	12	121
*Maxomys*	4	22	2	9.1	4	18	4	100.0	21	37

Total		1,566	83	5.3	1,407	90	1,048	74.5	392	277

The confidence level used for validating genotypes was fixed at 10^-3^.

## Discussion

Recent development of high-throughput genomic sequencing, considerably reducing the costs of sequencing, has opened up new perspectives for modern biology [45]. Here, we report a new laboratory procedure allowing the multiplexing and sequencing of one or several PCR amplicons for hundreds of samples in only one 454 run, allowing most of the reads generated to be attributed to their samples of origin. Data processing procedure and probabilistic model were developed for the validation of new variants and the estimation of confidence levels for each genotype produced. These procedures gave promising results, resulting in the successful genotyping of 1407 rodent samples from 24 different species, and the sequencing of 392 different variants of the *DRB *gene (exon 2) in only one half of a standard 454 picotiter plate. Replicates confirmed the high reproducibility (over 95%) of this genotyping method.

### Comparison with other methods

The method described here is particularly suitable for the determination of genotypes and sequences for highly variable and potentially multiplicated genes. For example, the high level of variant diversity observed for exon 2 of the *DRB *gene in *Myodes glareolus *(71 variants) prevents the use of classical methods like SSCP and DGGE. These methods are based on differences in migratory patterns during electrophoresis. As they do not directly provide information on the variant sequences, they are subject to homoplasy (i.e. the same pattern for different variants), thus preventing complete resolution for variants exhibiting a similar conformation or related sequences e.g. [46]. Nevertheless, these methods are useful for datasets containing a restricted set of variant forms e.g. in endangered species, [47,48]. They may be inadequate, however, for use in population genomics/genetics, involving large datasets of thousands of individuals. Indeed, in this study, it is unlikely that as many as 392 variants, or even the 71 variants identified for the bank vole *Myodes glareolus*, would have been distinguishable on the basis of their migratory patterns. Alternatives such as PCR-SSP, which is based on amplification using primers that are specific to a group of variants, were developed to overcome this technical constraint e.g. [20,21,49]. However, in addition to a poor resolution (variants within groups cannot be distinguished), PCR-SSP requires *a priori *knowledge of all the variant forms present in the population under study. As oligonucleotide chips, it is thus not suitable for most studies on non-model organisms. In our study, only 36 of 392 variants reported were already deposited in GenBank. Based on these observations, the reproducibility of our genotyping (estimated at 95%) was very satisfactory. The reproducibility of the classical methods described above is unlikely to ever approach this high value. Moreover, at least a part, if not all, of our failed results could be due to the low specificity of our primers for some variants, rather than to our genotyping method. Another disadvantage of using these indirect methods for variant characterization is the difficulties encountered to compare datasets generated in different laboratories, or in the same laboratory but at different times. Such comparisons require complementary techniques to relate the migration patterns, obtained using different machines or laboratories, to a given allelic form. Such limitations inevitably preclude meta-analyses. Lastly, indirect methods additionally require many PCR products to be sequenced in order to link the sequence to a particular migration pattern and thus to establish the migration patterns associated with particular allelic forms. In cases involving different allelic forms in the same individual, or in cases where the genes are duplicated or when selection favours heterozygotes [50], further manipulations are often needed before sequencing, such as gel excision or cloning. These additional manipulations are time-consuming and costly.

The 454 system overcomes most of these limitations by making the variant sequences directly available during genotyping. Furthermore, the variant does not need to be isolated before sequencing. Indeed, the 454 methodology includes emPCR, which separates the different DNA strains during the first processing steps. Consequently, the datasets can be studied without limitations concerning the number of variants to be detected and without any prior knowledge of the allelic forms present. Datasets from different laboratories may then be easily concatenated for the purpose of meta-analyses. This approach is suitable for genotyping individuals harboring high numbers of allelic forms. Other methods based on 454 system have been developed previously and show some similarities with ours. Babik *et al*. [31] carried out a 454 run to genotype 96 rodents *Myodes glareolus *at exon 2 of the *DRB *gene. They used 96 different tags and carried out 96 different purifications (i.e. as many tags and purifications as samples). By comparison, our method requires only a single purification step after DNA pooling, and a far smaller number of tags than samples for barcoding. Moreover we did not need to perform a library to ligate the adaptator prior to the emPCR. This reduces the number of steps (and so potential errors) during the laboratory experiment, and also greatly reduces the total cost of the experiment (probably of a third).

### Variants and genotype validation

In contrast to previous studies, our data processing relied on a probabilistic model to establish clear and objective thresholds for sequence and genotype validation. Other studies have described alternative criteria. Babik *et al*. [31] validated variants on the basis of their frequency within individuals. They considered variants with an observed frequency lower than 3% as artifacts, and thus removed them from the dataset. Variants were also validated based on their dissimilarity with the four most commonly found variant in a given sample. The validation score decreased when the similarity increased. This filter did not include the detection of chimeras, since chimeras are very dissimilar from both parent variants and may occur at a non-negligible frequency within individuals. In our study, we found chimeras to occur at a mean frequency of 6%. Such limitations in the variant validation procedure could partly explain the high number of variants per individual reported for *Myodes glareolus *in the previous study (up to 18 different variants validated in a single individual). Using our data validation procedure, chimeras are discarded in the last step involving sequence alignment and blast analyses. Another validation criterion used by Babik *et al*. [31] was the need for a sequence to occur in at least two different samples. This criterion could lead to the removal of many variants that are rare in populations. Such a bias may lead to misinterpretation of the principal mode of selection operating on the gene under study, or of the demographic tendencies of the population under study. Indeed, rare variants may be indicative of certain types of selection or demographic events. For example, population expansion, purifying selection or selective sweeps may result in an excess of rare variants [51,52]. Our variant validation procedure overcomes the problem of discarding rare variants because it analyzes each sample separately. Our procedure should also provide reliable results in cases where a given variant may be an artifact in one sample but a true variant in another sample. Another improvement of our validation process is that we describe a statistically based approach for determining a threshold (number of sequences per sample) for validating genotypes. The value of this threshold *T_2 _*may be redefined according to the number of copies of the amplified gene and to the ploidy level of the species studied. Our findings clearly illustrate this point. Indeed, the number of sequences per sample was sufficient for genotyping most of our rodents with the exception of *Maxomys *that displays multiple copies of the gene. Finally we recommend the systematic use of replicates for each 454 run, allowing the reproducibility of the genotyping procedure and, thus, the reliability of the run, to be estimated.

### Optimizing tagging and sample numbers

Our barcoding method led to 69% of the reads obtained being assigned to samples. This proportion is lower than the >95% reported by Binladen *et al*. [33] and Meyer *et al*. [32], who used alternative barcoding methods. The efficiency of our method could probably be improved by using a Titanium kit (Roche Diagnostics GmbH) that provide longer reads. We estimated that 9% of the reads that were unassigned were too short and did not encompass the full length of the sequence for exon 2 of the *DRB *gene. An estimated 8% of unassigned reads were attributed to the use of tags that form homopolymers GG with the key sequence (i.e. 2 of 24 reverse tag sequences could not be recovered as described above). It should thus be of utmost importance to exclude such tags from the experiment. Additionally, our criteria for the validation of reads in the first step of data processing may have been too stringent. The search for perfect matches with the complete primer sequences probably led to the elimination of a large number of reads. This criterion was established to discard reads that could present subsequent shifts in the reading frame of the tag (Table 2). Alternatively, a perfect match with only 5 to 10 bp of 5' sequences of the primers may have been sufficient to discard reads associated with reading frame shifts, and may have therefore allowed more reads to be assigned to samples. Finally, it may be possible to reduce the probability of incorrectly assigning reads to a sample due to sequencing errors within the tags. It is widely advised to use tags that differ by more than one substitution [53]. However, this reduces the number of combinations and thus the number of samples that can be multiplexed. Increasing the number of nucleotides within tags could counterbalance this limitation.

The probabilistic model that we developed may be used for optimizing the 454 run. When designing a 454 run, it is important to take into account the fact that intcreasing the number of samples results in fewer reads being retrieved for each of them. The optimal number of samples to be multiplexed for genotyping with a given level of confidence can be determined by simulating data before the run. The number of copies in the genome for the gene of interest must first be fixed, as well as the confidence level required. The model will then generate the number of sequences required per sample to give this confidence level. The number of reads guaranteed by the provider divided by the number of sequences generated by the model will then give the optimal number of samples to be multiplexed.

## Conclusions

We have described a method for barcoding and multiplexing hundreds of PCR-amplicons using half of a 454 plate. We were able to reassign about 69% of the reads generated to their sample of origin. The high number of reads obtained for each sample then allowed the genotype of each sample to be generated. We then used a probabilistic model allowing variant validation and attribution of a confidence level for each genotype based on several objective criteria. Using this approach, we obtained genotypes for exon 2 of the *DRB *gene in 1,407 samples from 1,374 rodents, belonging to 24 species. The *DRB *gene is a highly variable coding gene that may be duplicated several times in mammalian genomes.

This method may be improved in several ways. First, using longer tags may increase the proportion of reads that are finally assigned to their sample of origin, increase the number of samples multiplexed and decrease the risk of misassignment. Second, the number and order of the different filters (i.e. Steps 1 to 4 of our processing) may be modified and threshold values (*T_1 _*and *T_2_*) adapted to specific studies and genes. Here we applied three different filters that are very different by nature. The first filter (during Step 1) is related to the occurrence of variants within the whole dataset. It consists in withdrawing all the variants occurring only once in the dataset, which considering our criteria would not have been validated at the end of the process in any case. The second filter (during Step 2, *T_1_*) is related to the number of sequences yielded for each sample. It allows the elimination of samples (not variants) displaying too few sequences to be reliably genotyped according to our probabilistic model. The third filter (during Step 3, *T_2_*) is related to the number of sequences obtained for each variant within each sample. It consists in withdrawing variants that are present in low frequency within the samples. It should be noted that we decided to withdraw all variants occurring only once in the data set at the very beginning of our processing mainly for practical reasons. This considerably reduced the size of the dataset (13,671 variants could be withdrawn over a total of 19,847, i.e. a reduction of 69% in size of the data set) and greatly facilitated further manipulations. Yet this filter may be unnecessary because unique sequences will be in any case removed during Step 3. Besides we found logical to remove samples with too low numbers of sequences (that would not have been considered in the final set of genotypes in any case) before trying to discriminate true from artifactual variants within samples. This was done because our main objective was to get as many reliable genotypes as possible. This filter may not be optimal for other purposes, like acquiring as many variants (not genotypes) as possible for phylogenetic analyses, for instance. Lastly, the probabilistic model that we developed may allow the number of samples multiplexed in one 454 run to be optimized, as a function of the confidence level required for each genotype. The probabilistic approach proposed here may be improved in order to take into account biases in PCR-errors and yields. Our probabilities strongly depend on restrictive hypotheses (considering that all events have the same probability to occur whatever the nucleotide changes and sites considered). In this respect, our approach may provide null expectations for PCR-bias testing. Non-random processes in PCR-errors and yields like unequal probabilities of nucleotide changes, PCR-competition among variants and errors hotspots may (at least in theory) induce significant departures from our predictions. In our paper we provide a rough estimate of the probability of substitution errors based on our data (see Additional Information [Additional file 1]). More accurate estimates may result from inclusion of internal controls (i.e. variants of known sequences), which should be systematically incorporated in future experiments. Moreover, because most PCR-biases are expected to depend on data (i.e. organisms, genes, variants ...) as well as experiments (i.e. chemicals, laboratories ...), we think that more realistic models should further be based on the use of internal controls for model selection and parameter estimation. In the meanwhile we recommend compensating for such potential biases by choosing a high theoretical level of reliability for genotyping (99.9%) within our neutral model. Finally, an automated bioinformatics pipeline based on our stepwise procedure is currently being developed and will be available to other projects that may benefit from this genotyping approach. We believe that this methodology will be very useful for evolutionary and functional studies in the near future.

## Authors' contributions

The study was conceived and designed by MG, and was coordinated by NC and JFC. NC, GC and JFC performed the statistical analyses. MG and EG carried out the molecular biology procedures, the sequence alignment and the validation of the 454 sequencing data. GC devised the probabilistic program. MG and JFC wrote the manuscript. GC, EG and NC helped to draft the manuscript. All authors have read and approved the final manuscript.

## Supplementary Material

Additional file 1 **Additional Information**. Probabilities of observing artifactual sequences by substitution errors. Probability *f*(*r,m,n*) of observing at least *r *sequences of each of the *m *variants potentially observed for the *n *sequences of a given sample. Click here for file

Additional file 2 **Fig S1, Fig S2, Fig S3, Fig S6**. **Figure S1**. Histograms showing the number of sequences obtained for individual variants (*Nj*) for pools A and B. Insets display distributions for values below fifty. **Figure S2**. Histograms of the number of sequences obtained for each sample (*Ni*) after the first step of data processing for pools A and B. **Figure S3**. Confidence level for genotyping. *f *is the probability of amplifying, at least *r *times, all the different variants of the gene studied for a given sample. This probability depends on *n*, the total number of sequences by sample, and *m*, the maximal number of variants for the gene within a sample. *T_1 _*is the threshold value that corresponds to the minimal number of sequences required per individual to determine its complete genotype, with a 10^-3 ^probability of missing variants. Plots are given for different values of *r *= 1, 2, 5 and 10. **Figure S6**. The number of sequences obtained for each forward and reverse tag after the first step of data processing for pools A and B. Click here for file

Additional file 3 **Fig S4, Fig S5**. **Figure S4**. Histograms showing the distributions of *F_ij_*, the frequency of each variant *j *within each individual sample *i*. Data were grouped as a function of rodent genera. *m *is the maximal number of variants for the gene within a sample. **Figure S5**. True and artifactual variants of *DRB *exon 2 for a black rat (*Rattus rattus*). The two variants validated by our data processing are shown in green and blue, respectively. We obtained 36 sequences for the variant highlighted in green and 26 for the blue variant. Other variants are artifactual (n = 20). Variants corresponding to a mixture of blue and green correspond to recombinant chimeric sequences derived from a mixture of sequences of DRB*003 and DRB*006. Other artifactual variants corresponded to substitutions (sites in red, DRB*532, DRB*926, DRB*438, DRB*1412 and DRB*1636), indel (DRB*1653), pseudogenes orthologous to *RT1-Hb *(DRB*462) and the paralog *DQB *(DRB*1008). Click here for file
