# Supplementary material for: A 454 multiplex sequencing method for rapid and reliable genotyping of highly polymorphic genes in large-scale studies
Source: BMC Genomics. 2010 May 11;11:296. doi: 10.1186/1471-2164-11-296 (PMC2876125; doi:10.1186/1471-2164-11-296)
Supplement: Additional file 1 — Additional Information. Probabilities of observing artifactual sequences by substitution errors. Probability f(r,m,n) of observing at least r sequences of each of the m variants potentially observed for the n sequences of a given sample. [file 1471-2164-11-296-S1.DOC]

**ADDITIONAL INFORMATION**

***Probabilities of observing artifactual sequences*** ***by substitution errors***

Taking the probability of substitution errors arising during 454 sequencing to be *p*, the probability of at least one substitution error arising in a sequence of length *l* corresponds to $1-\left(1-p\right)^{l}$. Two values for *p* were considered. First, Huse *et al*. [e.g. 37] estimated *p*=0.08 %. Second, we made a very rough estimate using our data. To that purpose we used 1519 sequences yielded from 18 samples that were found homozygous for the same variant (pool B). 154 sequences were affected by at least one substitution. We can thus tentatively derive a substitution rate of 0.065%. Although slightly different, both estimates remain within the same order of magnitude. Given that the *DRB*-exon 2 fragment size amplified in this study was 171 bp, the probability of a sequence occurring with at least one substitution error in our dataset was 0.128 (for Huse’s estimate for *p*) and 0.104 (with our estimate for *p*).

The probability of the same substitution error occurring twice (i.e. the same mutated nucleotide at the same bp position) was determined for two homologous sequences with *l* independent sites and *b* different states (i.e. *b*=4 nucleotides in our case) for each site. The probability that *x* homologous sites experience the same mutation event is$\pi=(b-1)\left(\frac{p}{b-1}\right)^{x}$. We can then consider that the probability of observing *x* identical mutated sequences in the data set is $1-\left(1-\pi\right)^{l}$. In our case, the probability to observe three identical mutated sequences is 9.7 x 10^-9^ (for Huse’s estimate for *p*) and 5 x 10^-9^ (with our estimate for *p*).

***P*robability *f(r,m,n)* of observing at least *r* sequences of each of the *m* variants potentially observed for the *n* sequences of a given sample**

This probability depends on *n*, the total number of true sequences obtained for the sample, and *m*, the maximal number of variants for the gene within a sample. The value *m* depends on the number of copies of the gene and on the degree of ploidy of the studied organism. For instance, *m* = 2 for a nuclear gene present as a single copy within a diploid genome, and *m* = 4 for a nuclear gene duplicated within a diploid genome.

In the following we will only consider the case “when each variant occurs only once in the genome”. We considered this probability because we wanted to rule out the possibility to miss a variant in the genotype and because we had no prior knowledge on the samples that could have the same variant present in several copies in their genome. The probability to miss a variant that occurs more than once is necessarily lower than the probability to miss a variant that occurs only once. The probability we gave is thus conservative for samples that exhibited several copies of the same variant in their genome, which provided more guarantees to define full genotypes.

The model considers a subset of *n* sequences, randomly and independently drawn from an infinite set of sequences generated by the replication of a gene within the genome of a given individual. It also considers that the genome harbours *m* copies of the gene under study. The probability that a sequence comes from any given copy is constant. The relative proportion of sequences corresponding to the various variants is the same as that in the individual. In other words, we assume no replication bias according to the different variants or copies. The probability that a sequence comes from a given copy *i* is

$p_{i}=\frac{1}{m} \sum_{i=1}^{m} p_{i}=1$.

Let *K_i_* be the number of sequences coming from the copy *i* from *n* sequences, and let *K* be the vector with components *K_i_*:

$K=\left(K_{1},\ldots,K_{m}\right)$.

The vector *K* follows a multinomial distribution with parameters *n* and *m* defined by the probability function

$$\begin{matrix} \Pr(K_{1}=k_{1},\ldots,K_{m}=k_{m}) & = & \frac{n!}{k_{1}!\ldots k_{m}!}p_{1}^{k_{1}}\ldots p_{m}^{k_{m}} \\ & = & \frac{n!}{k_{1}!\ldots k_{m}!}\left(\frac{1}{m}\right)^{n} \end{matrix}$$

where

$\sum_{i=1}^{m} k_{i}=n$.

This model can then be used to determine the probability of having each of the copies represented at least *r* times among the *n* randomly drawn sequences. *r* is fixed. In other words, we want each copy to be represented at least *r* times within the subset of *n* sequences.

Let $E_{r,m}^{n}$ be the set of the different, possible situations. $E_{r,m}^{n}$ is the set of *K* values that respect the following two constraints:

$$\begin{matrix} \sum_{i=1}^{m} K_{i}=n \\ K_{i}>=r \text{for each copy }i \end{matrix}$$

The probability is then given as

$\begin{matrix} f(r,m,n)\text{ }= \Pr(E_{r,m}^{n}) & = & \sum_{K\in E_{r,m}^{n}} \Pr(K) \\ & = & \sum_{K\in E_{r,m}^{n}} \frac{n!}{k_{1}!\ldots k_{m}!}\left(\frac{1}{m}\right)^{n} \\ & = & n!\left(\frac{1}{m}\right)^{n}\sum_{K\in E_{r,m}^{n}} \frac{1}{k_{1}!\ldots k_{m}!} \end{matrix}$ (1)

The number of summed terms increases rapidly as a function of *r* and *n* because the number of elements of $E_{r,m}^{n}$is equal to

$\left|E_{r,m}^{n}\right|=\left(\begin{matrix} m+n-mr-1 \\ m \end{matrix}\right)$.

This poses computational problems that can be solved in our case because *m*, *n* and *r* usually have low numerical values. We limited ourselves to values $m\leq8$ and $r\leq10$.

The value of *n* was then fixed by the level of $\Pr(E_{r,m}^{n})$required.

We developed a program to compute *f(r,m,n)* =$\Pr(E_{r,m}^{n})$. This program gives, for fixed *m* and *r* values, the value of $\Pr(E_{r,m}^{n})$ for every *n* such that $\Pr(E_{r,m}^{n})\leq0,9999$. This program is freely available, as a Windows executable file (.exe) and as source code (.cpp), at the website <http://www.lirmm.fr/~caraux/Bioinformatics/NegativeMultinomial/>. It is written in generic ANSI-compatible C++ code, which can be compiled on most systems. This program was used to draw the graph shown in Figure 2, allowing the number *n* of sequences that need to be observed to be estimated, as a function of *r* and *m* and at a given confidence level.
