# Supplementary material for: A 454 multiplex sequencing method for rapid and reliable genotyping of highly polymorphic genes in large-scale studies
Source: BMC Genomics. 2010 May 11;11:296. doi: 10.1186/1471-2164-11-296 (PMC2876125; doi:10.1186/1471-2164-11-296)
Supplement: Additional file 2 — Fig S1, Fig S2, Fig S3, Fig S6. Figure S1. Histograms showing the number of sequences obtained for individual variants (Nj) for pools A and B. Insets display distributions for values below fifty. Figure S2. Histograms of the number of sequences obtained for each sample (Ni) after the first step of data processing for pools A and B. Figure S3. Confidence level for genotyping. f is the probability of amplifying, at least r times, all the different variants of the gene studied for a given sample. This probability depends on n, the total number of sequences by sample, and m, the maximal number of variants for the gene within a sample. T1 is the threshold value that corresponds to the minimal number of sequences required per individual to determine its complete genotype, with a 10-3 probability of missing variants. Plots are given for different values of r = 1, 2, 5 and 10. Figure S6. The number of sequences obtained for each forward and reverse tag after the first step of data processing for pools A and B. [file 1471-2164-11-296-S2.PPT]

## Slide 1
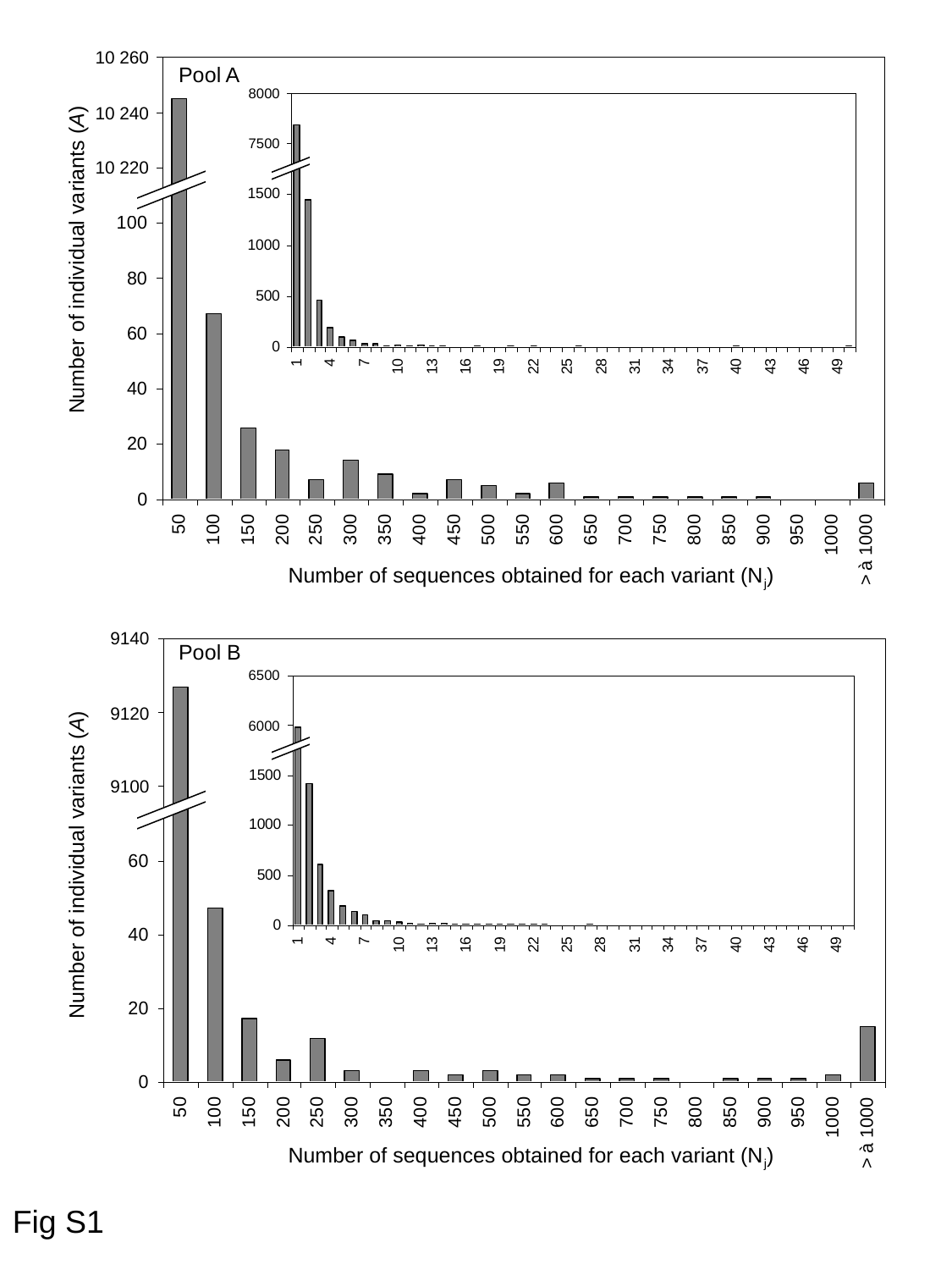

Pool A
8000
7500
9140
9120
9100
Pool B
6500
6000
10 260
10 240
10 220
Number of individual variants (A)
Number of sequences obtained for each variant (Nj)
Number of individual variants (A)
Number of sequences obtained for each variant (Nj)
Fig S1

## Slide 2
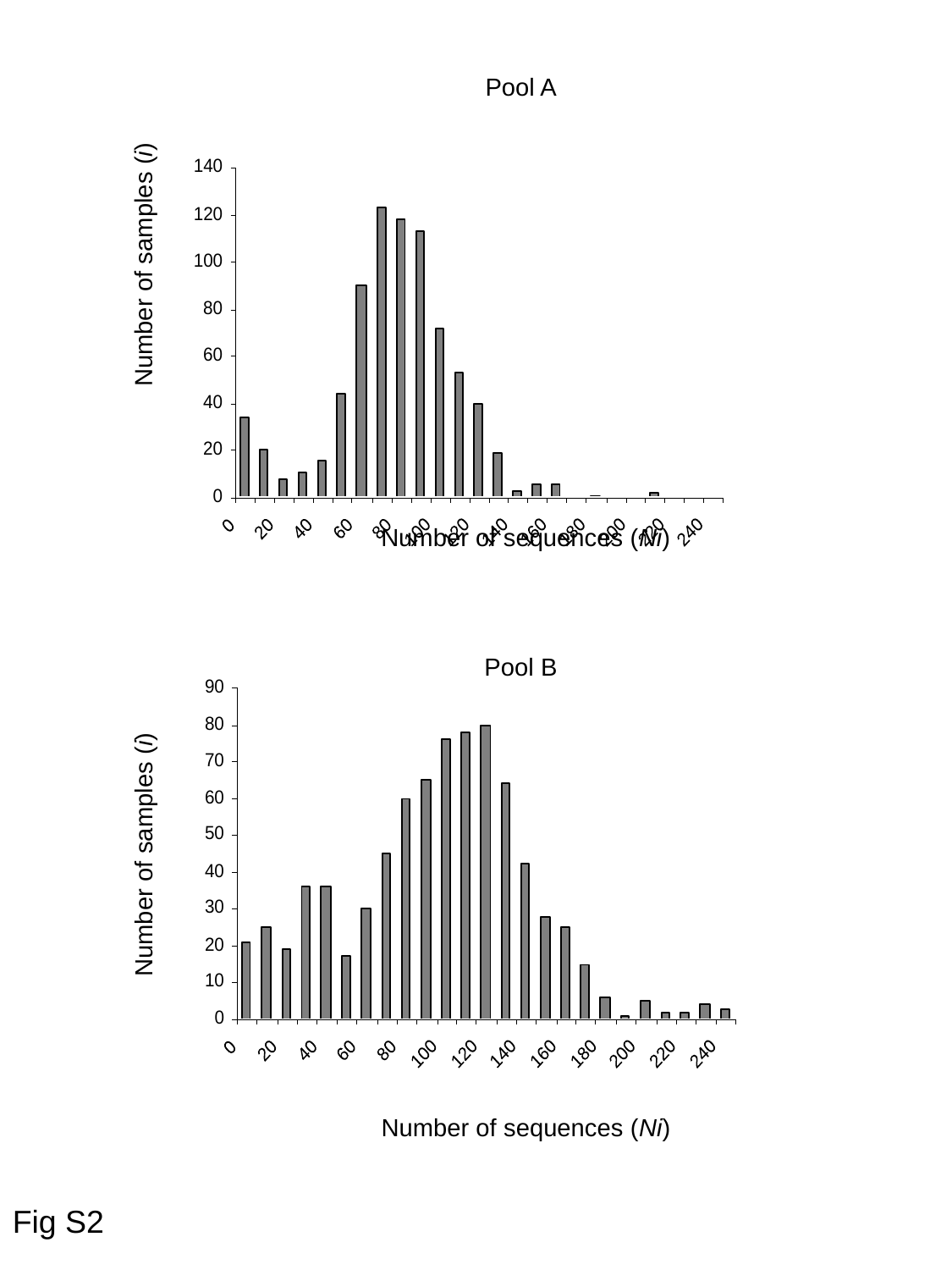

Pool A
Number of samples (i)
Number of sequences (Ni)
Pool B
Number of samples (i)
Number of sequences (Ni)
Fig S2

## Slide 3
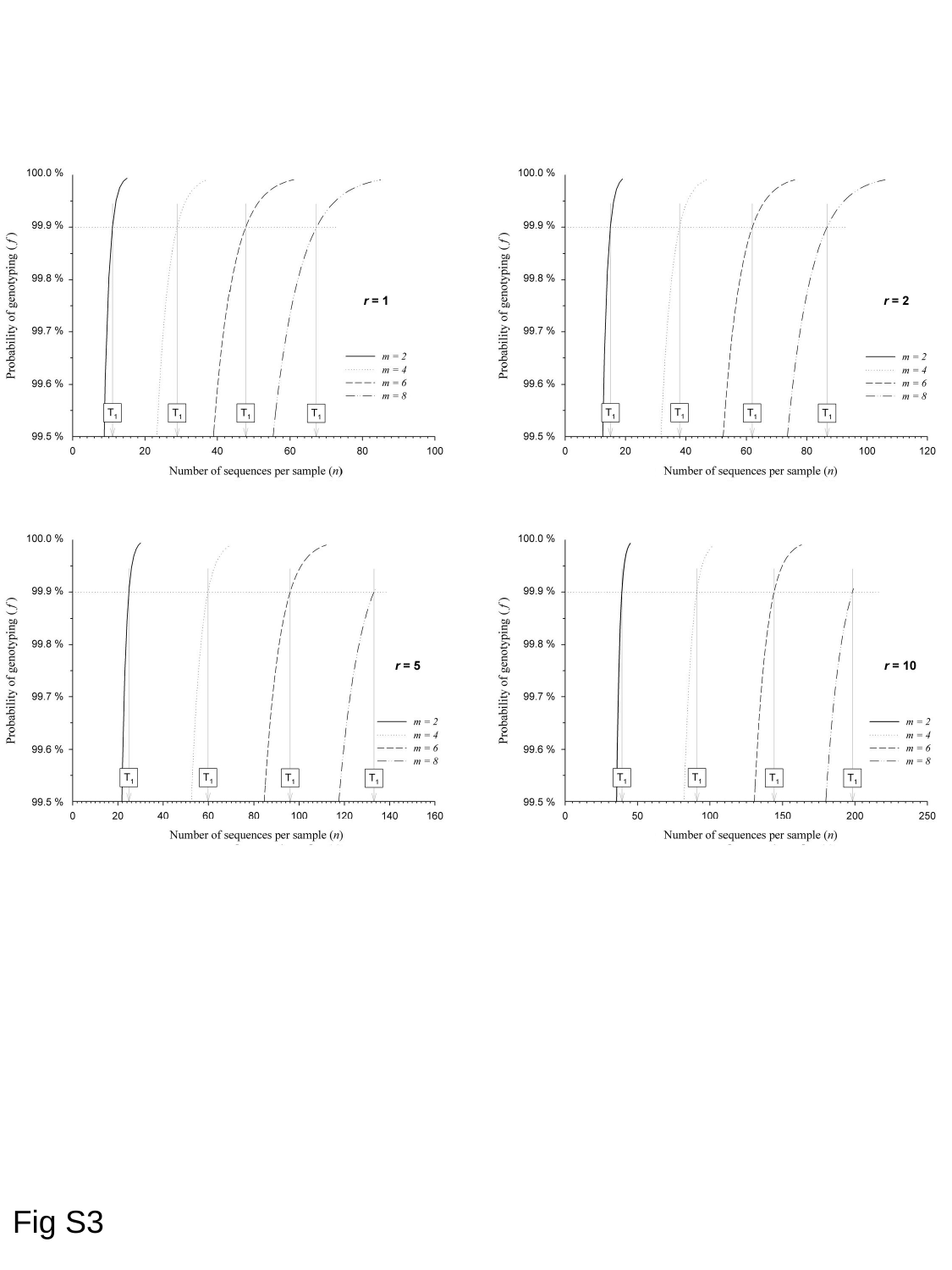

Fig S3

## Slide 4
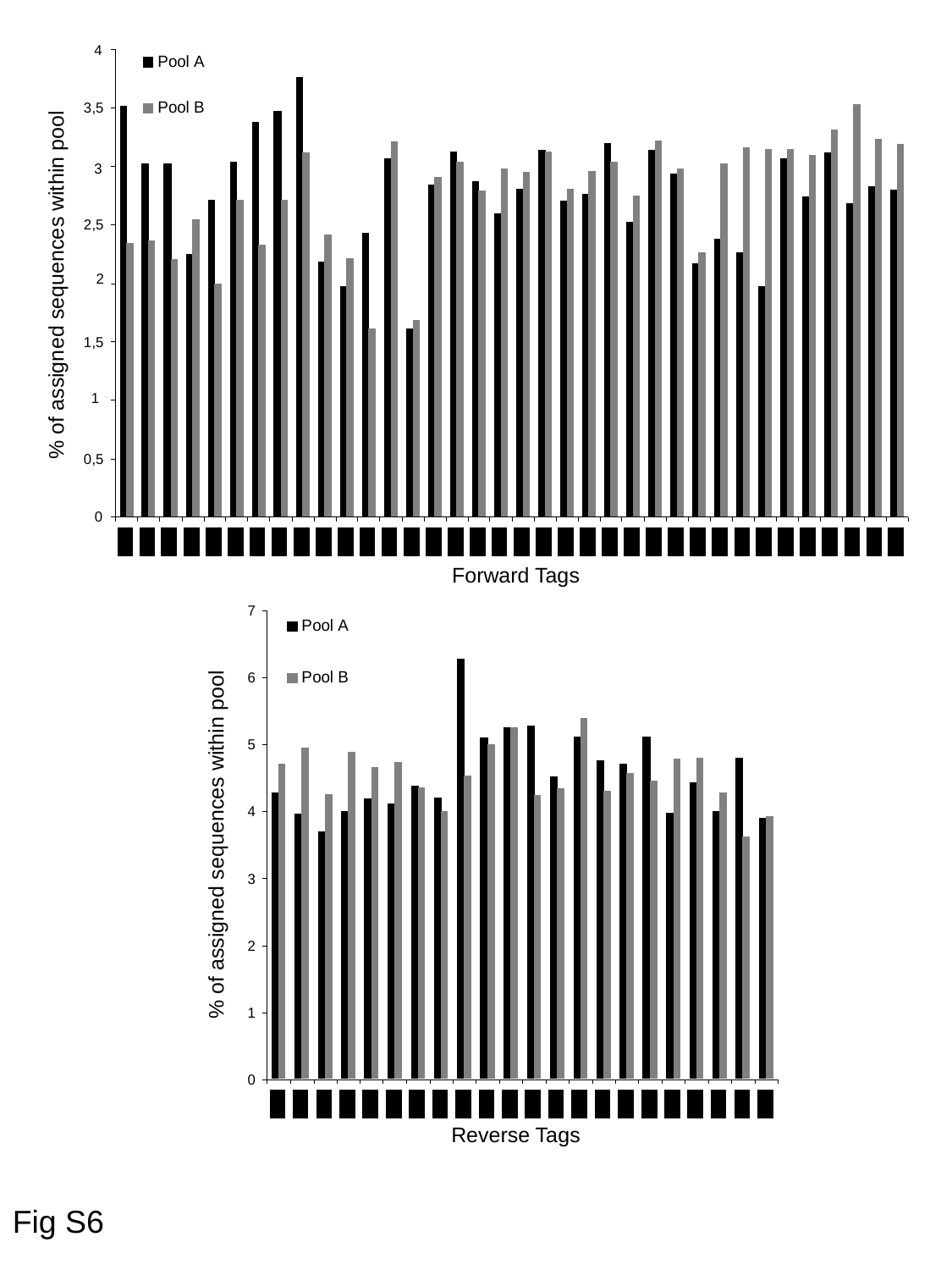

% of assigned sequences within pool
Forward Tags
% of assigned sequences within pool
Reverse Tags
Fig S6
