# Supplementary material for: A 454 multiplex sequencing method for rapid and reliable genotyping of highly polymorphic genes in large-scale studies
Source: BMC Genomics. 2010 May 11;11:296. doi: 10.1186/1471-2164-11-296 (PMC2876125; doi:10.1186/1471-2164-11-296)
Supplement: Additional file 3 — Fig S4, Fig S5. Figure S4. Histograms showing the distributions of Fij, the frequency of each variant j within each individual sample i. Data were grouped as a function of rodent genera. m is the maximal number of variants for the gene within a sample. Figure S5. True and artifactual variants of DRB exon 2 for a black rat (Rattus rattus). The two variants validated by our data processing are shown in green and blue, respectively. We obtained 36 sequences for the variant highlighted in green and 26 for the blue variant. Other variants are artifactual (n = 20). Variants corresponding to a mixture of blue and green correspond to recombinant chimeric sequences derived from a mixture of sequences of DRB*003 and DRB*006. Other artifactual variants corresponded to substitutions (sites in red, DRB*532, DRB*926, DRB*438, DRB*1412 and DRB*1636), indel (DRB*1653), pseudogenes orthologous to RT1-Hb (DRB*462) and the paralog DQB (DRB*1008). [file 1471-2164-11-296-S3.PPT]

## Slide 1
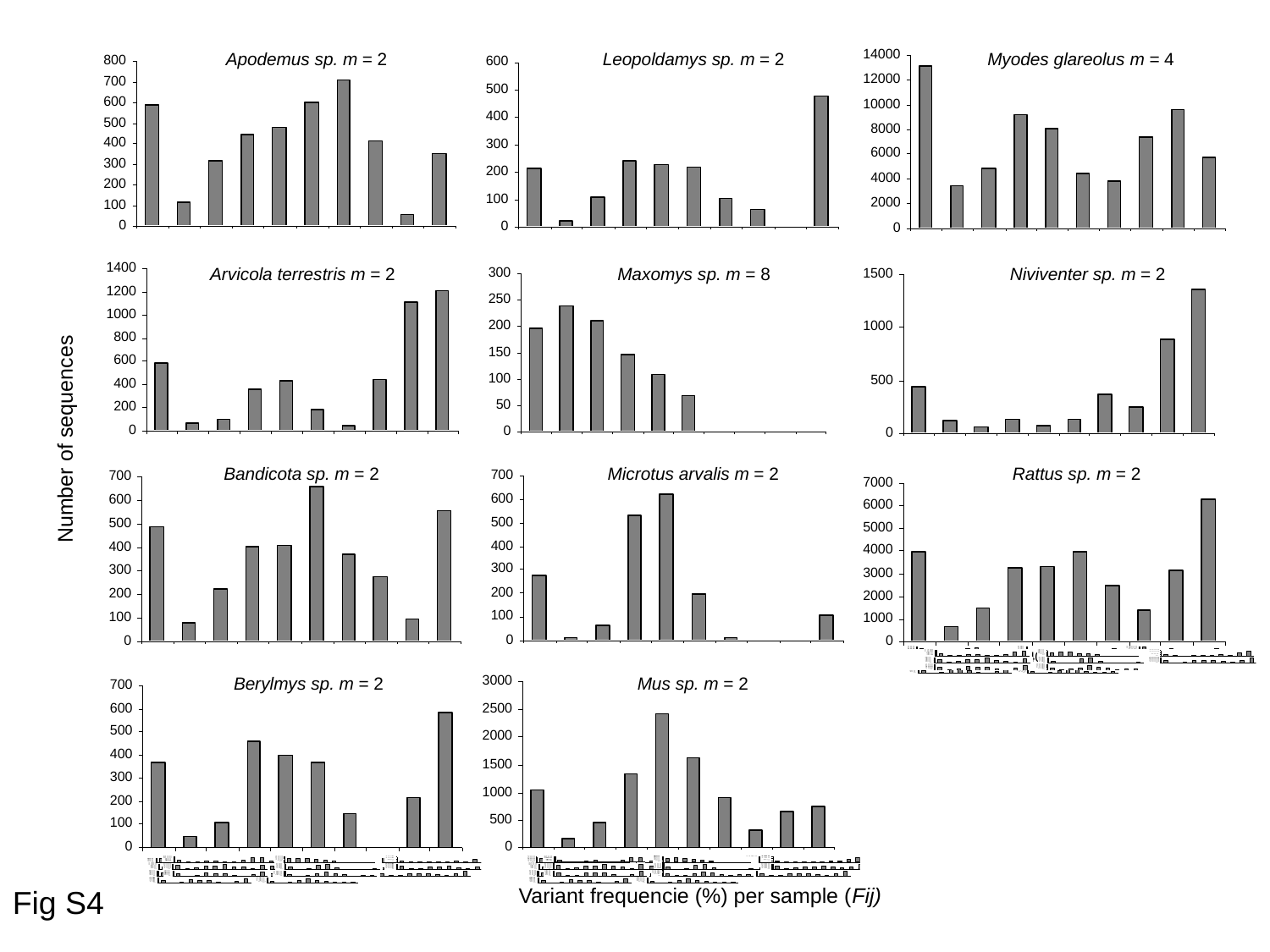

Apodemus sp. m = 2
Leopoldamys sp. m = 2
Myodes glareolus m = 4
Arvicola terrestris m = 2
Maxomys sp. m = 8
Niviventer sp. m = 2
Number of sequences
Bandicota sp. m = 2
Microtus arvalis m = 2
Rattus sp. m = 2
Berylmys sp. m = 2
Mus sp. m = 2
Fig S4
Variant frequencie (%) per sample (Fij)

## Slide 2
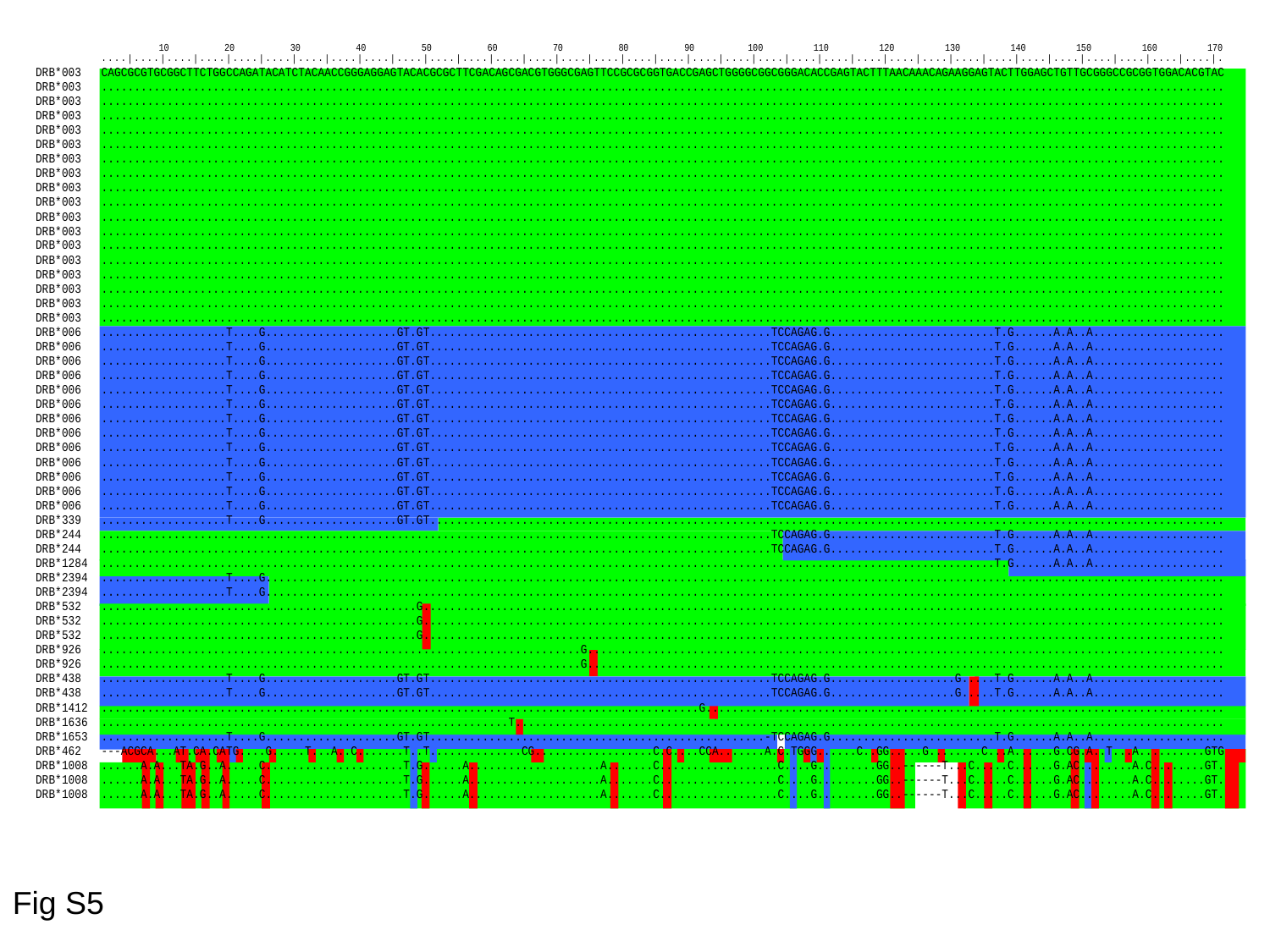

Fig S5
